# Protein expression in experimental malignant glioma varies over time and is altered by radiotherapy treatment

**DOI:** 10.1038/sj.bjc.6603190

**Published:** 2006-05-30

**Authors:** C Wibom, F Pettersson, M Sjöström, R Henriksson, M Johansson, A T Bergenheim

**Affiliations:** 1 Department of Oncology, University Hospital, SE 901 85 Umeå, Sweden; 2 Research Group for Chemometrics, Department of Chemistry, Umeå University, SE 901 87 Umeå, Sweden; 3 Department of Neurosurgery, University Hospital, SE 901 85, Umeå, Sweden

**Keywords:** glioma, proteomics, SELDI, rat, multivariate analysis

## Abstract

Radiotherapy is one of the mainstays of glioblastoma (GBM) treatment. This study aims to investigate and characterise differences in protein expression patterns in brain tumour tissue following radiotherapy, in order to gain a more detailed understanding of the biological effects. Rat BT4C glioma cells were implanted into the brain of two groups of 12 BDIX-rats. One group received radiotherapy (12 Gy single fraction). Protein expression in normal and tumour brain tissue, collected at four different time points after irradiation, were analysed using surface enhanced laser desorption/ionisation – time of flight – mass spectrometry (SELDI-TOF-MS). Mass spectrometric data were analysed by principal component analysis (PCA) and partial least squares (PLS). Using these multivariate projection methods we detected differences between tumours and normal tissue, radiation treatment-induced changes and temporal effects. 77 peaks whose intensity significantly changed after radiotherapy were discovered. The prompt changes in the protein expression following irradiation might help elucidate biological events induced by radiation. The combination of SELDI-TOF-MS with PCA and PLS seems to be well suited for studying these changes. In a further perspective these findings may prove to be useful in the development of new GBM treatment approaches.

Despite significant efforts, the outcome of treatments for glioblastoma (GBM) has so far been disappointing. After a multimodal approach including surgery, postoperative radiotherapy and chemotherapy the median survival is still only 14 months (Stupp *et al*, 2005). Therefore, there is an urgent need to improve conventional treatment modalities and/or develop new ones. However, several problems are associated with the development of new approaches for treatment of GBM.

One problem is that more rapid methods are needed to evaluate the biological effects of conventional glioma treatment modalities in new combinations. Today the results of treatment are usually evaluated radiologically several months after treatment, which is unsatisfactory for a fast growing tumour like GBM. Likewise, new methods are of importance in the development of new, target specific therapies, where treatment efficacy may be detected by molecular means before radiological response. Moreover, there is a need to improve the knowledge regarding pathophysiology in the treatment of glioma.

Recently, new experimental techniques utilising mass spectrometry (MS) for analysing protein expression patterns in various tumour conditions have given us new knowledge in human tumours (Wiesner, 2004; Reyzer and Caprioli, 2005). In malignant glioma it is possible to distinguish between tumour and nonmalignant brain tissue, as well as between different grades of glioma, using direct-tissue analysis by matrix-assisted laser desorption/ionisation (MALDI) MS and subsequent cluster analysis (Schwartz *et al*, 2004). To date, however, the main focus of studies employing proteomics has been to detect biological markers for early diagnosis of disease. For example, specific serum protein patterns have recently been detected in patients with different grades of astrocytomas (Liu *et al*, 2005). Similar results have also been obtained in studies of carcinoma in prostate (Adam *et al*, 2002; Banez *et al*, 2003), breast (Vlahou *et al*, 2003) and ovaria (Petricoin *et al*, 2002).

In the present investigation we have utilised surface enhanced laser desorption/ionisation time of flight mass spectrometry (SELDI-TOF-MS) (Merchant and Weinberger, 2000) to detect changes in protein patterns in tumour tissue following radiotherapy in an experimental rat glioma model.

There is a wide range of different data analysis approaches that could be applied to this type of mass spectrometric data, for example neural networks (NN) has successfully been applied to SELDI data (Ball *et al*, 2002; Liu *et al*, 2005) as well as support vector machines (SVM) (Koopmann *et al*, 2004) and hierarchical clustering (Poon *et al*, 2003). The data acquired in this study were analysed using the multivariate projection methods principal component analysis (PCA) and partial least squares – discriminant analysis (PLS-DA). These analytical data projection methods have proved to be powerful tools for analysing multivariate data obtained in diverse biomedical studies using a wide range of methods, including separation techniques such as high performance liquid chromatography (HPLC), gas chromatography (GC) and liquid chromatography (LC), spectroscopic methods, for example, nuclear magnetic resonance (NMR) and MS, and combinations of separation and spectroscopic methods such as GC/MS and LC/MS. For example, in an early study Wold *et al* (1982) used PCA to discriminate between normal and brain tumour tissues as described by 105 GC peaks, with only a few observations in each group. More recently, PCA and PLS have been commonly applied as data analysis tools in metabonomics studies of complex data structures obtained in NMR, GC/MS and LC/MS analyses (Adam *et al*, 2002; Eriksson *et al*, 2004).

## MATERIALS AND METHODS

### Rat glioma model

The syngenic BT4C rat glioma model was used for this investigation. Briefly, the BT4C rat glioma model is a transplacentaly nitroso-urea-induced rat tumour, previously characterised as a gliosarcoma (Bergenheim *et al*, 1994; Johansson *et al*, 2000). BT4C cells growing in log phase were suspended in Dulbecco's modification of Eagle's MEM (DMEM) (GIBCO, Paisley, Scotland) supplied with 5% BDIX rat serum to a concentration of 2 × 10^4^ cells in 5 *μ*l. Inbred BDIX rats were anesthetised with a 1 : 1 mixture of Hypnorm® (fluanisone 10 mg ml^−1^ and fentanyl citrate 0.315 mg ml^−1^) and Dormicum® (Midazolam 5 mg ml^−1^) of which 0.5 ml was given per 100 g rat. Xylocain (10 mg ml^−1^) was used for local anaesthesia under the scalp before incision. Using a stereotactic technique, 5 *μ*l of the cell suspension was implanted 3.5 mm to the right of bregma at a depth of 4.5 mm into the right caudate nucleus. Special care was taken to prevent cell reflux through the burr hole, which was finally covered with bone wax. During the implantation procedure cells were kept on ice and viability was monitored by intermittent Tryphan blue staining. After implantation animals were housed in a controlled environment with 12 h light/dark cycles and provided food and water *ad libitum*. Animals were monitored by an experienced animal keeper during the whole length of the experiment.

A total of 24 animals were implanted and divided into two groups of 12. One group received radiotherapy delivered as a 12 Gy single fraction 12 days after tumour implantation, while the other served as an untreated control group. Radiotherapy was given as whole brain irradiation using a conventional 4 MV linear accelerator. The dose was based on previous experiments, indicating that 12 Gy single fraction has equivalent tumour growth inhibitory effect as 4 Gy × 5 fractionated irradiation (data not shown). Three animals from each group were killed 1, 5, 7 and 12 days after irradiation. Tumour tissue was carefully dissected from each animal and snap frozen. In addition the contralateral normal frontal brain was collected and frozen. Tissue was stored at −80°C until analysed.

Regarding animal welfare consideration was taken not to expose animals to unmotivated suffering. The experiments were carried out in strict accordance with the UKCCCR guidelines (Workman *et al*, 1998). The experiments were approved by Umeå University's animal research ethics committee in accordance with the Swedish Animal Welfare Act 1988:534 as last amended by SFS 2002:550, which are adopted in consequence of EC Directive 86/609/EEC.

### Tissue handling

Tissue samples of approximately 0.2 g. were thawed and homogenised using a Dounce Tissue Grinder (Kimble/Kontes, Vineland, NJ, USA) in 400 *μ*l homogenisation buffer 1 (100 mM HEPES, pH 7.4, 100 mM NaCl, 0.5% CHAPS). Homogenisation buffer 1 (0.8 ml) was added to the homogenate and the mixture was allowed to incubate on ice for 30 min before centrifugation at 20 000 r.p.m. for 20 min in 4°C. The supernatant was collected and two volumes of protein-denaturing buffer (8 M urea, 1% CHAPS, PBS) were added. The mixture was incubated on a shaker at 4°C for 30 min, then snap frozen in liquid nitrogen and stored at −80°C. The pellet fraction was rehomogenised in 400 *μ*l homogenisation buffer 2 (5 M Guanidine–HCl, 50 mM Tris (pH 8.0), 0.5% CHAPS) and incubated on ice for 3 h before centrifugation at 20 000 r.p.m. for 20 min at 4°C. The new supernatant fraction was collected and diluted in two volumes homogenisation buffer 2, snap frozen and stored at −80°C. Both homogenisation buffers contained EDTA-free protease inhibitor (Roche Applied Science, Indianapolis, IN, USA). The protein content of each sample was determined using the BCA (bicinchoninic acid) assay (Pierce Biotechnology Inc., Rockford, IL, USA) and the mean of three individual readings was used to determine the appropriate dilution required for the SELDI analysis.

### SELDI-TOF-MS

**Figure 1:**
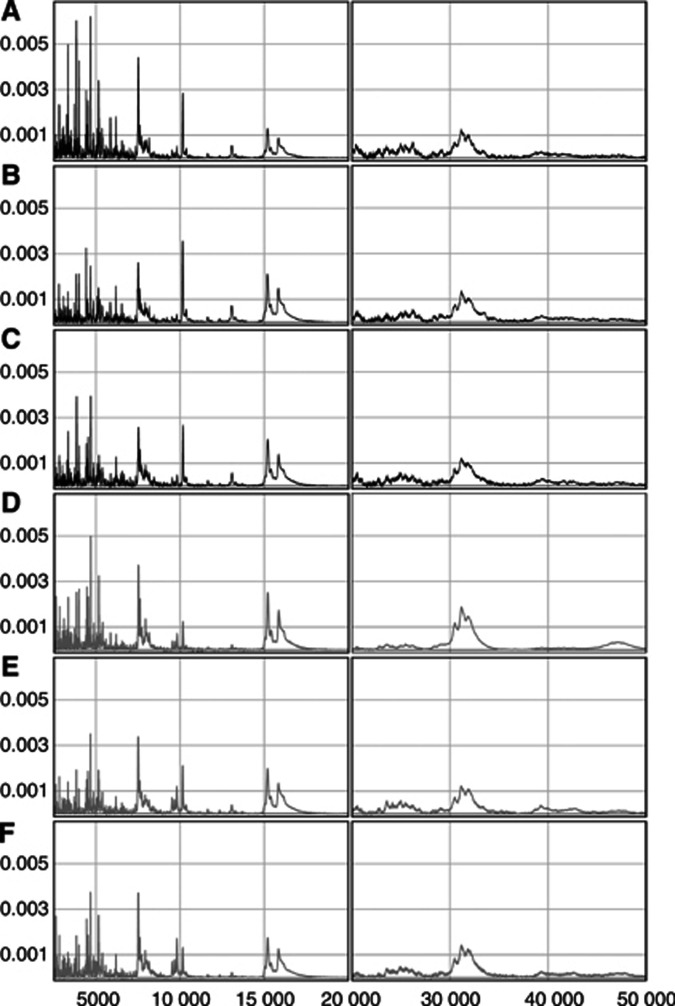
Representative protein profiles from six different tumour samples, all taken 12 days after irradiation. The *y* axes denote the relative normalised intensity and the *x* axes the different *m/z* variables. The different mass regions for each sample are plotted in separate panels, low mass (2.5–20 kDa) to the left and high mass (20–50 kDa) to the right. The top three panels show profiles from animals in the untreated control group (**A**–**C**) and the bottom three profiles from radiotherapy-treated (12Gy in a single fraction) animals (**D**–**F**).

Surface enhanced laser desorption/ionisation is characterised by its selective binding of a subset of all proteins in a given sample to a ProteinChip® (Ciphergen Biosystems Inc., Fremont, CA, USA). The bound proteins are subsequently analysed in the TOF mass spectrometer, yielding a specific protein profile for each individual sample (Figure 1).

A number of different types of array and binding buffers were initially examined for this experiment. An IMAC30 array and a phosphate binding buffer (0.1 M PO_4_, pH 7.5, 0.5 M NaCl) was found to be the most suitable combination, providing higher spectrum quality and a larger number of peaks than the other tested options. A Biomek 2000 Laboratory Automation Workstation robot (Beckman Coulter Inc., Fullerton, CA, USA) was utilised for all array preparation and sample application steps in combination with a MicroMix5 shaker (Diagnostic Products Corporation, Los Angeles, CA, USA), which was employed for all array incubation steps, set to program 5 and amplitude 20.

IMAC30 arrays were assembled into a Bioprocessor (Ciphergen Biosystems Inc.), charged with Zn^2+^ by adding 50 *μ*l 100 mM ZnSO_4_ and left to equilibrate for 5 min at room temperature. The Bioprocessor was subsequently emptied and the metal charging step was repeated. To remove unbound metal ions the arrays were then washed twice with 100 *μ*l 1 mM HEPES for 5 min. Thereafter, the arrays were equilibrated three times with 100 *μ*l binding buffer for 5 min, after which the samples were applied. Samples from one fraction at a time were thawed and diluted in binding buffer to a concentration of 0.15 *μ*g *μ*l^−1^ in a 96-well plate on ice, mixed on a MicroMix5 shaker for 2 min then transferred to the Biomek 2000. Of each diluted sample (100 *μ*l) was added to the Bioprocessor and allowed to incubate for 1 h. All samples were processed and applied to the arrays in triplicates, according to a completely randomised scheme. To facilitate assessment of the method reproducibility, identically treated standard quality control (QC) samples were added onto a number of random spots per Bioprocessor. In order to remove unbound sample the arrays were then washed with 150 *μ*l binding buffer three times for 5 min, followed by two 1 min washes with 150 *μ*l 1 mM HEPES. Before matrix addition the Bioprocessor was dismantled by removing the top and gasket and the arrays were air dried. Thereafter, two deliveries of 1 *μ*l saturated sinapinic acid (Ciphergen Biosystems Inc.), diluted in 50% acetonitrile and 0.5% trifluoroacetic acid, were added to each spot on the arrays.

Arrays were analysed in a TOF mass spectrometer (PBS-II; Ciphergen Biosystems Inc.). Mass spectra were generated in positive ion mode using an accelerating voltage of 20 kV. Each spot was analysed twice, with individual settings optimised for different mass regions. To analyse the lower mass region (2–20 kDa), the laser intensity was set to 210 and 215 for the pellet and supernatant fraction samples, respectively, the detector sensitivity to 7 and the focus lag times was 782 ns. To analyse the higher mass region (20–50 kDa), a laser intensity of 260 and a detector sensitivity of 10 was used in conjunction with a focus lag time of 1328 ns for both fractions. Mass spectra were acquired by averaging the data from 192 laser shots fired at 16 different positions within each spot. Different positions were used to acquire high and low mass spectra. The mass spectrometer was externally calibrated using an all-in-one-protein standard (Ciphergen Biosystems Inc.).

### Data analysis

Spectral data were acquired using SELDI-TOF-MS. Baseline subtraction was performed on all spectra using Ciphergen Proteinchip® Software. Data from both fractions (supernatant and pellet) were then exported as raw spectra files in csv format. For the high mass region, data in the interval 20–50 kDa were exported for multivariate analysis. We applied a lower limit cutoff of 2.5 kDa to the low mass region to avoid all matrix-related noise, and hence exported data in the interval 2.5–20 kDa for multivariate analysis. All multivariate analyses were carried out using Evince 1.1 (Evince for PC, Mac and Linux, Umbio: Umbio AB, C/O Uminova Innovation AB, Box 7978, 907 19 Umeå, Sweden).

The QC samples were used to evaluate the method reproducibility, and thus were treated separately. All low mass range spectra generated from the QC samples were normalised together in the interval 2.5–20 kDa using the Ciphergen ProteinChip® Software. The program was set to calculate noise within the same mass region and peak clusters to be compared were then automatically selected, utilising the Biomarker Wizard tool in Ciphergen Proteinchip® Software with the following settings; first pass signal to noise ratio=5, min peak threshold=50% of all spectra, cluster mass window=0.3% of mass, second pass signal to noise ratio=2. Information on the detected peak clusters was finally exported in.csv format for reproducibility calculations.

### Data preprocessing for multivariate analysis

The values for the different *m/z* variables were not consistent between the many different spectra. For meaningful comparison of many spectra, each has to be built up by the same set of well-defined variables. Therefore, using a binning procedure, each spectrum was transformed into 5000 variables, to be analysed by multivariate projection methods such as PCA and PLS-DA. All bins corresponded to small mass intervals (3.5 and 6 Da for the low mass and the high mass data, respectively) represented by the mean value of the intensities therein. This process also had a smoothing effect on the spectra. Each spectrum was then normalised individually by dividing all of the variables in it by the total intensity of the spectrum. Before the multivariate analysis data were then transformed (centred) by subtracting each variable by its mean.

### Principal component analysis

The central idea of PCA is to extract a few, so-called, principal components describing as much as possible of the variation present in the data. The principal components are linear combinations of the original variables and uncorrelated to each other. Here, **t** represents the scores vectors and **p′** the loadings vectors for each component. *A* is the number of principal components and **E** is the residual matrix.

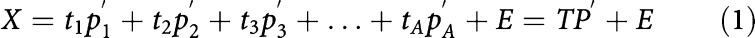


The principal components can be determined using the NIPALS algorithm (Wold, 1966) or singular value decomposition (SVD) (Jolliffe, 1986). The scores (**t**) show how the objects and experiments relate to each other. Objects close to each other in a score plot are similar to each other in terms of the variables that influence the plotted components. The loadings (**p**) reveal the variables that are important for the patterns seen in the score plot. By examining the scores and the loadings important groupings in the data can be identified and explained.

### Partial least squares

Partial least squares is a multivariate regression method that relates the data matrix (**X**, descriptors) to a *y* response that can be either single (**y**) or multiple (**Y**). Partial least squares has proved to be a powerful tool for finding relationships between descriptor matrices and responses, especially when there are more variables than observations and the variables are collinear to each other and noisy. The PLS theory and methods discussed here concern single *y* responses. As in PCA, principal components are constructed to reduce the dimensions of **X**. In order to obtain the principal components, PLS maximises the covariance between the response variable **y** and a linear combination of the original variables **t**=**Xw**, where **t** is the score vector, **X** is the data matrix and **w** is the weight vector. A more detailed description of PLS can be found elsewhere (Garthwaite, 1994; Burnham *et al*, 1996, 1999; Wold *et al*, 2001).

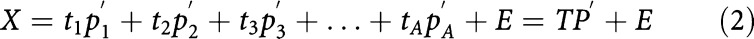


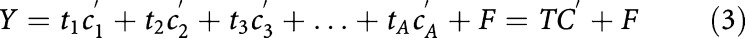

**t**=score vector for **X**; *A*=the number of PLS components; **p**=loading vector for **X**; **c**=loading vector for **Y**; **E**=residual matrix for **X**; **F=**residual matrix for **Y**; When using PLS for discriminant analysis (PLS-DA), dummy variables are used to describe the classes to which different samples can be assigned. This is performed by creating pairs of binary variables for each class, for example ones and zeros, where a one signifies that the object belongs to the class concerned, and a zero implies that it does not. With a PLS-DA model it is possible to predict whether or not an object belongs to a specific class considering the predicted class variable. A predicted value greater than 0.5 implies that the sample belongs to that class and a value lower than 0.5 implies that it does not belong to it.

### Validation of models

To determine the ideal number of components to calculate and analyse in order to optimise the interpretability and predictive power of any multivariate model, it must be validated. Components that do not explain a significant amount of variation should not be analysed and interpreted since they may mainly describe noise. We choose here to restrict the analysis to principal components that had relatively large eigenvalues and visually appeared to describe variation related to the classification. For each component a *R*2*X* value was calculated, representing the percentage of variation described in the data. These values are presented for each PCA model.

In order to make valid predictions from a PLS model it is important not to overfit it, that is to avoid fitting it to random noise in the observed data. To ensure that this is avoided, appropriate validation techniques need to be applied. Crossvalidation, an example of an internal validation procedure used here, involves removing observations from the data in a stepwise procedure, computing a prediction model based on the remaining samples and testing the calculated model by comparing the estimated values with the empirically obtained values for the excluded observations. This process is then repeated by excluding a new selection of observations, until all observations have been excluded once. Finally, a prediction error term is calculated, the prediction error sum of squares (PRESS), defined as:

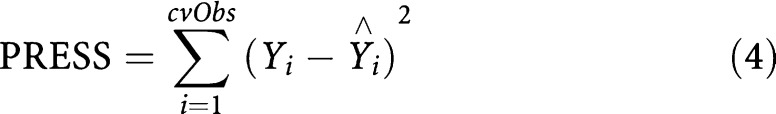
 
where *cvObs* is the number of observations in the test set, *Y*_*i*_ is the observed value for observation *i* in the test set and *Ŷ*_*I*_ is the predicted value for the same observation. PRESS is used, in turn, for calculating *Q*2*Y*:

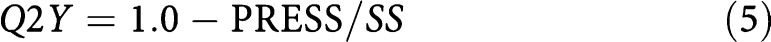
 
where SS is the sum of squares for *Y*. *Q*2*Y* can be described as the percentage of the response (*Y*) that will be correctly predicted for a set of new observations using the current model. Crossvalidation can be performed by removing one or many observations in every run. Removing one observation in each run is commonly referred to as the leave-one-out procedure. As our data contains replicate groups, we applied segmented crossvalidation in which entire replicate groups were excluded instead of one sample at a time, to avoid including data from the same replicate group in both the test- and training sets.

### Identification and analysis of regions of interest

In regions where differences between two compared groups were detectable by means of multivariate analysis, we wanted to further investigate them univariately. By studying the loadings from the multivariate analyses and relating them to the scores, one can find the most important variables for separating the groups. It has been shown that, for a single component PLS model, the first weight vector, **w1**, provides the best estimate of the importance of a given variable, for describing the response. Later components are only needed for correcting the predictions made by the first component for all the variation in the **X** matrix that is not correlated to the response, but still affects the prediction (Trygg, 2002). For example, *m/z* variables with high absolute **w1** values contribute heavily to the group separation. In this study we identified mass spectrometric regions of interest (ROI) as parts of the spectrum where the *m/z* variables had corresponding absolute **w1** values greater than 0.015 (ROI:∣w1∣>0.015) for low mass data and 0.010 (ROI:∣w1∣>0.010) for high mass data. The small number of samples did not allow the use of statistical permutation analysis to determine these cutoff limits (Johansson *et al*, 2003). These ROI were subjected to additional investigation, as follows. Within each ROI we identified protein peaks, defined as *m/z* variables where the mean protein profile curve had a local maximum. For any given ROI with positive **w1** values, peaks were identified in the mean spectrum derived from the spectra for the group with positive scores in the first component, that is the group with binary response 1 in the PLS-DA model. In ROI with corresponding negative **w1** values, the opposite mean spectrum was used to identify peaks, that is the mean spectrum derived from the spectra for the group with binary response 0. As the high mass regions of all protein profile curves displayed a large amount of small amplitude noise, they were smoothed before peak detection using a five point adjacent averaging algorithm. The relative peak intensity for all remaining peaks was extracted from every individual spectrum in both groups. To compensate for possible mass shifts, intensity values from individual spectra were selected as local maximum intensities (peaks) within ±0.2% of the *m/z* value defined as a peak in the mean spectrum. In cases where more than one peak was identified in the scanned mass interval, the one with the closest *m/z* value to that of the mean peak in question was extracted. The peak intensity values of the treated and the nontreated groups were then compared.

Since each of the groups consisted of samples from only three animals, each analysed in triplicate, we needed to confirm that observed differences between the groups were due to treatment effects and not to differences between animals. This was performed using a nested linear model with fixed effects comparing treatment effects with a full model allowing differences between rats. When there was little evidence of a difference between individuals (*P*>0.1), *t*-test statistics were applied to assess the differences between treated and control animals. Conversely, when there appeared to be a difference in peak intensity between different individuals (*P*<0.1), the differences between the two groups were investigated using a linear mixed effects model (lme). In this model not only the measurement error within each rat but also the sampling of individual rats was assumed to be a random effect while the treatment effect was assumed to be fixed. All univariate analysis was performed utilising the freeware R 2.1.1 (R Development Core Team. R: A language and environment for statistical computing. Vienna, Austria; 2005).

## RESULTS

### Protein profiling QC

Randomly spotted QC samples were analysed together with the rest of the samples to assess method reproducibility. For the pellet fraction a total of 12 QC samples were analysed together with the rest of the sample material in two Bioprocessors. Here, 16 peaks were selected and compared, resulting in a coefficient of variance (CV) of 20.1%. For the supernatant fraction 14 QC samples were analysed and16 peaks were selected with a resulting CV of 16.5%.

### Comparing normal and tumour tissues

The protein expression pattern from tumour tissue was compared with that from contralateral normal brain tissue, with the purpose of validating the strategy. Principal component analysis applied to each data set from the separate time points disclosed obvious separations between tumour samples and normal samples for all data sets (data not shown).

**Figure 2:**
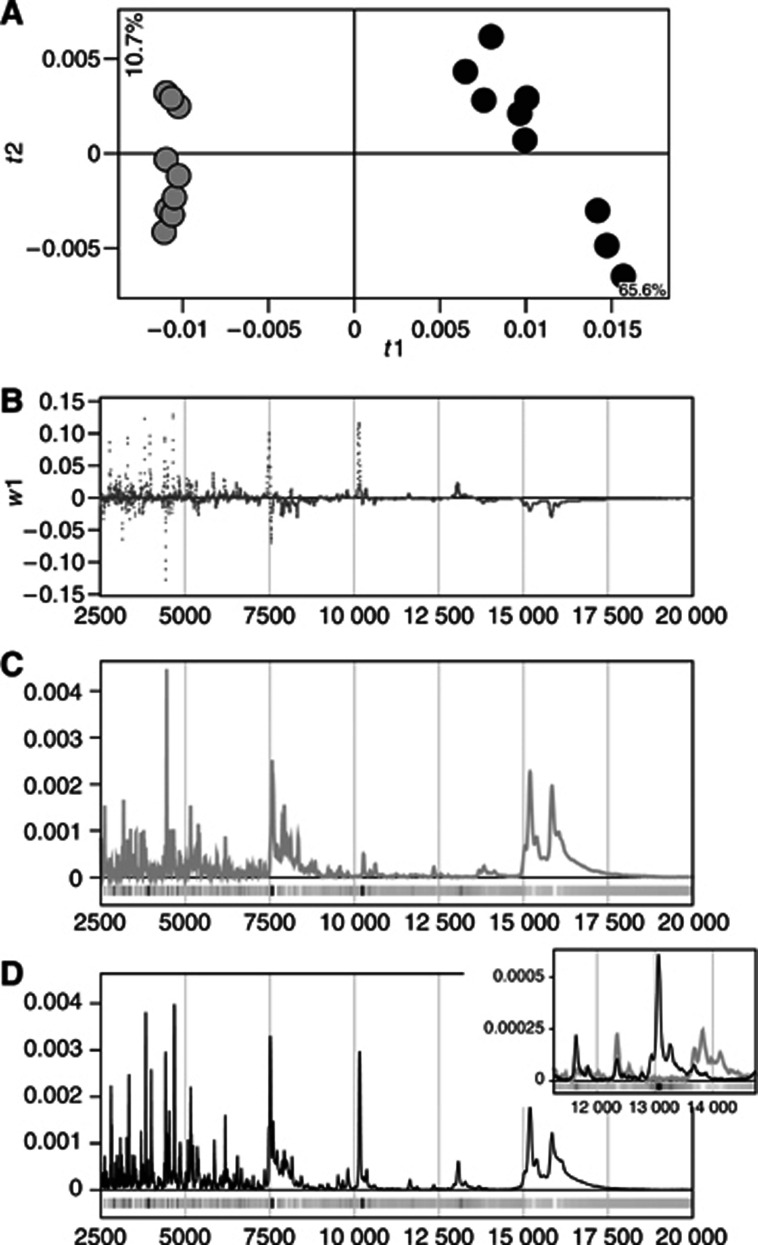
Partial least squares model based on low mass range (2.5–20 kDa) data from the supernatant fraction of three normal tissue (grey) and three tumour tissue samples (black). All samples are from the untreated control group, killed 24 days after implantation and analysed in triplicate. (**A**) Partial least squares scores **t1** *vs* **t2**. *Q*2*Y*(cum)=0.96 for the first two components. (**B**) First dimension PLS loadings (**w1**) plotted against mass variables. (**C**) Low mass range, mean spectrum derived from all nine spectra generated from the normal tissue samples. (**D**) Low mass range, mean spectrum derived from all nine spectra generated from the tumour tissue samples. The shading of the bars under the spectra in (**C**) and (**D**) indicates the sign and magnitude of the **w1** values for each *m/z* variable: black corresponds to high positive **w1** values (i.e. parts of the spectra where the tumour samples' spectra display substantially higher relative intensities than the normal samples' spectra), white corresponds to high negative **w1** values (i.e. parts of the spectra where the normal samples' spectra have substantially higher relative intensities) and grey corresponds to low **w1** values. The colour coding is the same in the blown-up region of the spectra (inset), but scaled according to the minimum and maximum **w1** values associated with that region.

Figure 2C and D show the mean spectra for the low mass region from both groups, derived from nine individual spectra from the supernatant fraction from untreated rats killed 24 days after implantation. Application of PLS-DA to the data yielded a complete separation of the two groups in the first dimension (Figure 2A). In Figure 2B the PLS-DA loadings (**w1**) are plotted for each variable on the *m/z* axis, to illustrate which variables, that is parts of the spectra, are the most important in the separation of the two groups. Variables on the *m/z* axis with a relatively high absolute **w1** value were more important for the separation than those with a relatively low absolute value, meaning that high absolute values in **w1** corresponded to *m/z* variables discriminating between the two groups.

### Outliers

Initially all data sets were analysed separately with PCA, and possible outliers were identified. Two single observations were removed, one from the day 7 data set and one from the day 12 pellet data set. The first one displayed very large residuals (see Eq. 1) and the second was discovered to be a clear outlier in score space. They both displayed protein profiles highly dissimilar to the other two observations in the triplicate set. Furthermore, two complete sample triplicates were removed since it was clear they belonged to a different data set, one from day 5 and one from day 7 after irradiation. All outliers were further examined by hierarchical clustering, and it was found that these observations positioned separately from the rest of the observations in each respective data set (data not shown).

### Changes in protein expression over time in treated and untreated control tissues

**Figure 3:**
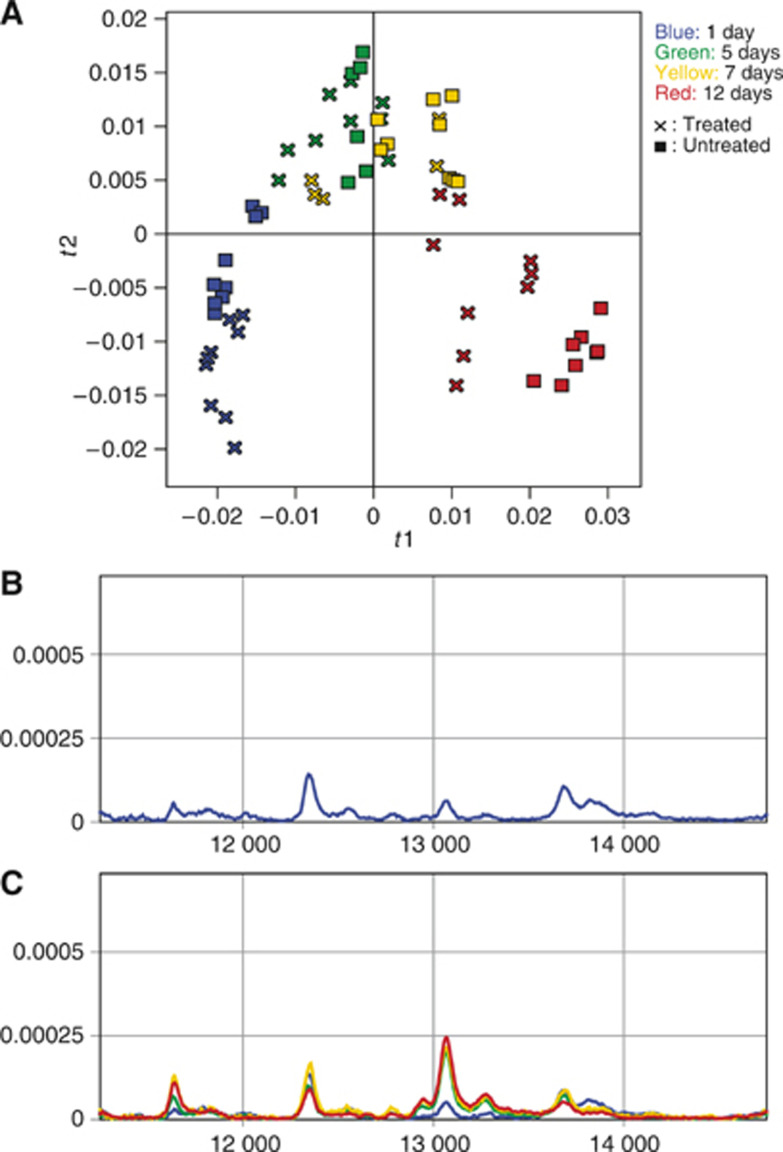
Principal component analysis reveals a time trend in protein expression during tumour progression (**A**). *R*2*X*(cum)=0.65 for the first two components. × and ▪ represent treated and untreated samples, respectively, and the different colours represent samples collected at different time points after treatment, as follows: blue=1 day; green=5 days; yellow=7 days; red=12 days. (**B**) and (**C**) display mean protein profiles within a specific spectral region, derived from untreated and treated samples, respectively. The samples are from the same time points as above and the same colour coding is applied.

Application of PCA to mass spectrometric data obtained from glioma tissues (both fractions and both mass ranges) from treated and untreated animals killed 1, 5, 7 and 12 days after irradiation revealed distinct time trends in protein profiles (Figure 3A), associated with the tumour's successive growth. There was also a clear distinction between treated and untreated samples from days 1 and 12, since the protein profiles from the untreated samples from day 1 display a closer resemblance to the protein profiles from day 5 than the treated ones. Similarly, the protein profiles from the treated samples from day 12 displayed a closer resemblance to the protein profiles from day 7 than the untreated ones. The profiles from days 5 and 7 displayed similar tendencies, but less clearly. Figure 3B and C illustrate how a few protein peaks develop over time in untreated (control) and treated samples, respectively.

### Treated *vs* untreated tumours

Comparing treated and untreated tumour samples from each individual time point reveals unambiguous separation between the classes for days 1 and 12 after irradiation. Data collected on days 5 and 7, however, does not separate the classes, although there are some weak tendencies towards separation present in some of the fraction subsets to these data (data not shown).

**Figure 4:**
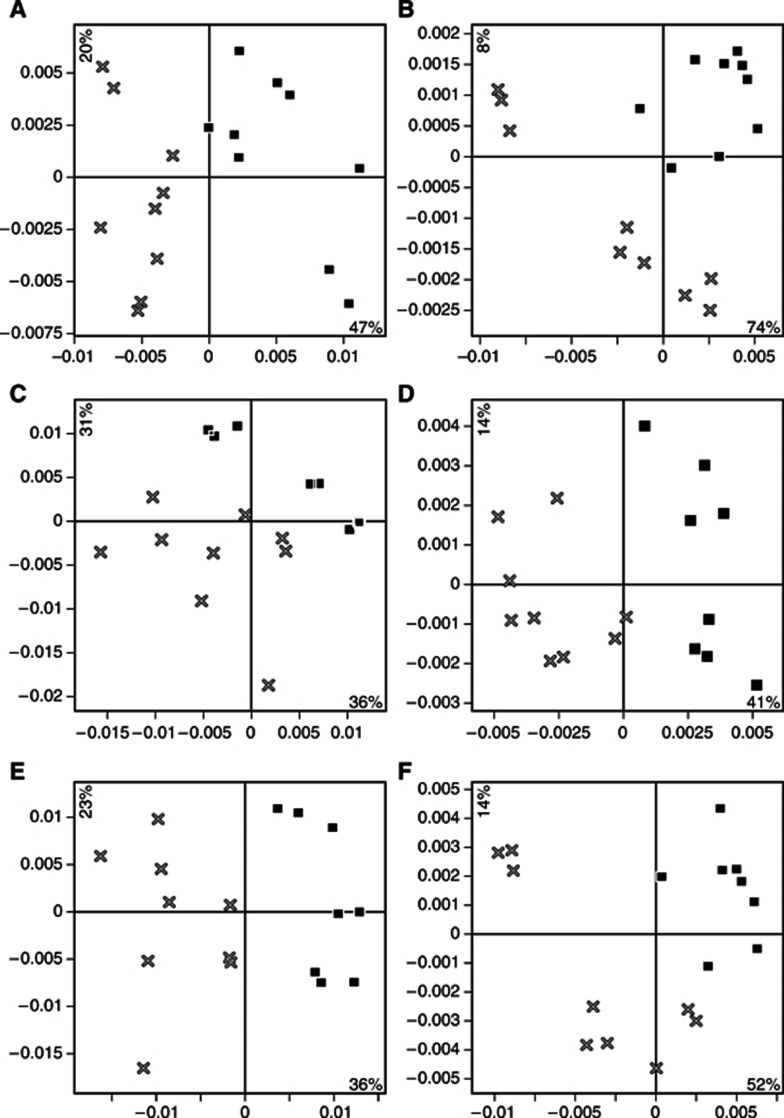
Principal component analysis of data collected 12 days after treatment from different fractions and mass regions clearly distinguishes treated and untreated tumour samples. All panels represent the first two PCA scores vectors plotted against each other, **t1** on the *x* axes and **t2** on the *y* axes. × and ▪ represent treated and untreated samples, respectively. (**A**) Supernatant, low mass region (2.5–20 kDa). (**B**) Supernatant, high mass region (20–50 kDa). (**C**) Pellet, low mass region. (**D**) Pellet, high mass region. (**E**) Pellet and supernatant, low mass region. (**F**) Pellet and supernatant, high mass region.

Samples from both the treated group and the untreated control group collected on day 12 after irradiation (24 days after implantation) were investigated by PCA to detect differences in complete protein profiles related to treatment effects. Data sets from the different mass ranges were analysed separately and data sets from the two fractions were treated both separately and together (Figure 4). The treated and control samples could be separated in all data sets analysed. Therefore, discriminating information was obtained throughout the whole mass spectra, as well as in both the analysed protein fractions.

### External validation of PLS-DA

**Figure 5:**
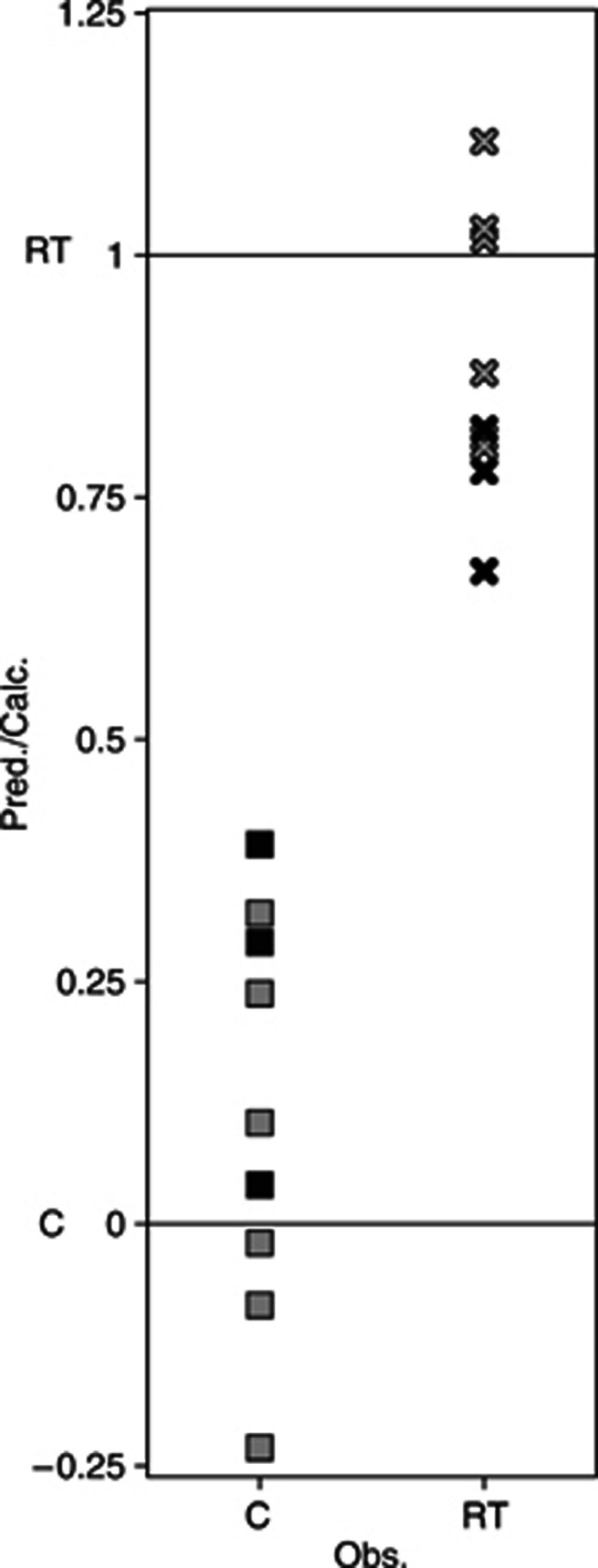
External validation of a PLS-DA model of the effect of radiation treatment based on data from tumour samples collected 12 days after treatment. A predicted value close to 1 implies that the sample belongs to the radiation-treated group (×), based on the protein profile of its low mass range supernatant fraction and a predicted value close to 0 implies that it belongs to the control group (▪). All samples in the external test set (black) and the calibration set (grey) were correctly classified using the single-component PLS-DA model. *Q*2=0.83.

The PLS-DA model based on the low mass data set from the supernatant fractions of samples collected 12 days after treatment modelling the differences in protein profiles between irradiated and nonirradiated control samples, was externally validated. A test set consisting of six samples, one sample triplicate from the control group and one sample triplicate from the irradiated group, were removed from the modelling step. A PLS-DA model was calculated based on the remaining samples, and the model was then used for predicting whether the samples in the test set were irradiated or not, based on their protein profiles. As can be seen in Figure 5 all samples were assigned to the correct group: an observed value of 1 representing irradiated samples and a value of 0 control samples.

### Analysis of ROI

**Figure 6:**
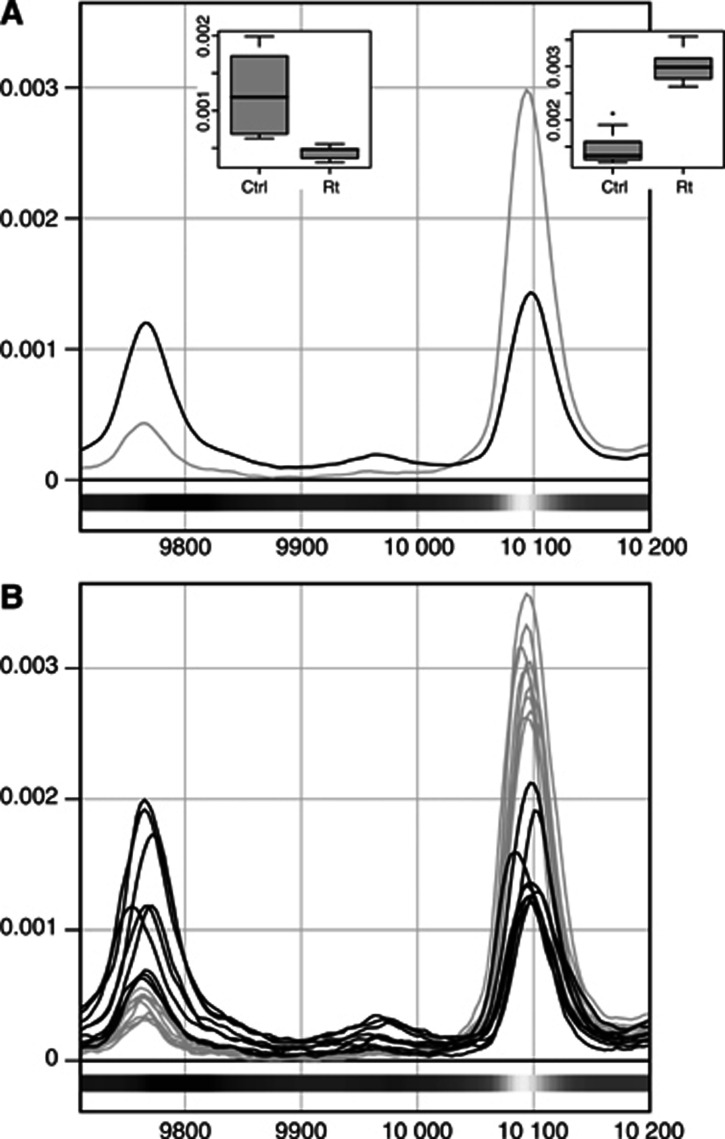
Two spectral ROI, defined as segments where *m/z* variables displayed absolute **w1** values greater than 0.015 (for low mass regions). (**A**) Spectral image of two ROI, plotting the mean spectra from samples collected in the supernatant fraction 12 days after treatment, comparing treated (grey) and untreated (black). (**B**) Same two ROI as in (**A**) with all individual samples plotted. The shading of the bars under the spectra indicates the sign and magnitude of the **w1** values (see Figure 2) derived from the PLS-DA model that underlies the localisation of ROI. The right-hand peak in both panels corresponds to peak 10147.5 in Table 2. There was no clear evidence that the left-hand peak was significantly altered by radiotherapy, so it is not listed in the table.

Partial least squares models were calculated for each of the separate data sets from day 12 after irradiation (see Table 1). The previously described peak identification strategy was applied to the variable loadings from these models. In the supernatant fraction we found 31 peaks, out of a total 47 peaks in the ROIs, in the low mass region that displayed significantly different expression patterns after therapy. In the high mass region the corresponding numbers were 12 out of 35 peaks. For the pellet fraction we found that 17 out of 37 peaks in ROIs showed significantly differentiated expression patterns in the low mass region, and 17 peaks out of 28 in the high mass region (Table 2 lists peaks with a significantly altered level of expression and Figure 6 gives a spectral image of two ROI).

## DISCUSSION

The results of this study show that high throughput mass spectrometric protein profiling, combined with multivariate analysis methods such as PCA and PLS-DA, are excellent methods for investigating and comparing protein expression from brain tissue extracts. The data revealed distinct differences between radiotherapy-treated and untreated glial tumours in an experimental glioma animal model. Significant differences were also detected between glioma and normal brain, which is consistent with previous studies on both glioma (Zhang *et al*, 2003; Iwadate *et al*, 2004) and other types of tumours (Melle *et al*, 2004). It is obvious that our method permits tumour progression to be monitored over time, since protein profiles from different time points during tumour growth formed subgroups in score space when modelled with PCA. These findings clearly suggest that the tumour protein expression pattern in the BT4C model successively and systematically changes over time. Moreover, by analysing animals subjected to radiotherapy in the same manner, it becomes apparent that this systematic development is changed by radiotherapy treatment (Figure 3). To our knowledge, this is the first study indicating that it may be possible to follow treatment responses to radiotherapy with a high throughput mass spectroscopic method applying multivariate statistical methods.

It is well known that protein expression profiles differ both between tumours with different histopathologic grades (Ware *et al*, 2003; Iwadate *et al*, 2004) and between different tumour stages (Cheung *et al*, 2004; Roblick *et al*, 2004). However, to our knowledge, the finding that the tumour protein expression profile changes over time in histologically identical tumours is novel. This may be due to molecular changes in the tumour cell biology *per se*, but changes in the tumour–host interaction may also contribute. In our experience, however, the BT4C cells grow homogenously in the rat brain without any obvious visible changes in the cellular distribution within the tumour, until late in the course of progression when necrosis may appear. Nevertheless, necrosis is generally not present at the time points when tissues were sampled in this study (Bergenheim *et al*, 1994) and radiotherapy alone has only a growth inhibitory effect without altered morphology (Johansson *et al*, 1999). Therefore, the observed changes in protein expression were not clearly correlated to gross morphological changes in the tumours. For this study we found it most important to account for this variation of protein expression over time in our comparative analyses. Thus, all comparisons between tumours were performed on tissues harvested at the same time point after implantation.

In the present study, we found clear differences between irradiated and nonirradiated glioma tissue. In total, summing the findings in all separate data sets, 77 peaks were found to have significantly altered expression levels 12 days after radiotherapy treatment. These proteins or protein fragments may include potentially interesting markers of treatment outcome. The majority of significantly changed peaks were observed in the low mass regions of the mass spectra and it may be suggested that many of these peaks are fragments of larger functional proteins. To date, there is little work performed in glioma focused on finding markers for early tumour detection (Liu *et al*, 2005), and in the general field of oncology there are only a few studies on finding markers for quantification of treatment efficacy (Gadducci *et al*, 2004; Reyzer *et al*, 2004). Such a marker could be of considerable clinical importance. Among the protein peaks we identified that helped to distinguish irradiated tumours from nontreated tumours, some could be involved in the pathogenesis of clinically observed side effects (Costello *et al*, 2004; Swennen *et al*, 2004) while others may represent protein markers that could be used to evaluate therapeutic efficacy. However, to establish these peptides as markers for therapy response, samples from individuals with differential responses should be investigated. So far, no marker for radiation-induced cytotoxicity in malignant glioma has been identified and clinically established.

The candidate biomarkers for treatment effects described herein need to be further characterised to gain additional insight into the biology of radiotherapy. Such knowledge may be valuable for understanding the pathogenesis of brain tumours and, ultimately, how to treat them. Furthermore, it would be of great importance to detect similar protein expression differences in more clinically accessible compartments, such as CSF, serum or microdialysis samples, they could then be rapidly and reliably analysed as treatment efficacy markers. This could take us one step closer to the ultimate possibility of individualising treatment modalities and monitoring the effects instantly, especially with regard to all of the novel drugs that now are introduced in the clinic (Krause and Van Etten, 2005). The use of protein expression profiling may also be important in neuro-oncology for the subclassification of tumours (Furuta *et al*, 2004; Schwartz *et al*, 2004; Liu *et al*, 2005), as a complementary approach to the morphological and genomic methods currently used (Kleihues and Ohgaki, 1997).

In the BT4C glioma, tumour progression involves changes in the overall protein expression profile, as shown in this study (Figure 3). Both treated and untreated control tumour samples displayed clear time trends when modelled with PCA. Interestingly, at two of the time points measured (1 and 12 days after irradiation) there were clear differences between protein profiles from treated and untreated control samples. In both instances, the untreated samples seemed to have progressed further than their radiotherapy-treated counterparts. More specifically, protein expression profiles from untreated samples from day 1 showed greater resemblance than treated samples from the same time point to the day 5 protein profiles. In much the same way, treated samples from day 12 displayed greater similarity to protein profiles from day 7 than the untreated samples from the same time point. Treated and untreated control samples from days 5 and 7 displayed the same tendencies, but much less clearly. Taken together these findings could indicate that progression of protein expression in tumours was slower in treated samples than in untreated samples.

By applying multivariate analysis techniques we were able to use all our data for analysis. In doing so we circumvented the difficulty of selecting peak clusters for analysis in the initial investigation. Instead, we used insight gained from the initial multivariate analysis to select ROI from which all possible protein peaks were selected for further univariate analysis. This is in marked contrast to other studies in which peaks were selected before any comparative analysis was applied (Adam *et al*, 2002; Vlahou *et al*, 2003; Liu *et al*, 2005). In Figure 2 both of the mean spectra display prominent peaks at about 7500 Da which, judging by the loadings, strongly influence the PLS model. A valid question is therefore whether the observed difference in masses for these particular peaks is real or an artefact related to instrument drift over time. It is, however, reasonable to assume that the observed difference is in fact real, since all of the samples were analysed simultaneously and in a randomised fashion, which should minimise the occurrence of such artefacts. This hypothesis was also confirmed by extracting the exact *m/z* values for each individual spectrum in the two groups and comparing them between the groups with a *t*-test, which indicated that the peaks had significantly different masses (data not shown).

As emphasised above, multivariate projection methods were essential statistical tools in this investigation. Principal component analysis was used to assess the similarity of different samples according to their protein profiles and to detect groupings. A data set with *n* variables can theoretically be visualised by plotting the observations in an *n*-dimensional space. However, it is impossible for us to view data efficiently in more than three dimensions because of the cognitive limitations of the human mind. Principal component analysis is an unsupervised method, which tries to find the directions in the multidimensional space that explains most of the variation in the data. The PLS method is similar to PCA but is supervised in the way that it tries to optimise the correlation between the principal components with the response. The response can be either continuous or discrete and PLS models can be used for predictive purposes. If the response is discrete and binary the modelling procedure is called PLS-DA. The previously described methods have useful properties for analysing proteomics data. They are well suited for analysing data where the number of variables greatly exceeds the number of observations. They also deal efficiently with noisy and correlated variables, which are often observed in spectral data. In the machine-learning community it is generally accepted that a sample to feature ratio (SFR) of at least 5–10 is required for robust classification. For mass spectra in a biomedical context SFRs are typically in the range 
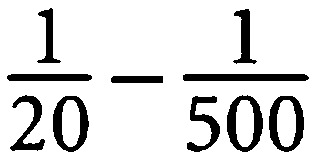
. In machine-learning approaches such as neural networks the conventional solution is to reduce the feature space dimensionality by variable selection (Somorjai *et al*, 2003). The multivariate methods described and used herein rely on linear algebraic operations and are transparent in contrast to neural nets (for example), which are often seen as black boxes. Thus, the influence of specific variables in the models can be evaluated, facilitating the identification of peaks and ROI.

One of the shortcomings of the present study is that the number of animals in each group was relatively small. From a data analysis perspective it is always preferable to have a larger number of observations. On the other hand, since the study focused on a controlled, inbred animal model in which all individuals are basically genetically identical, we believe that the differences between the different groups studied should outweigh the individual variations within the groups. We also took appropriate measures to adjust for possible individual effects when the results were statistically compared, in order to avoid erroneous conclusions. Based on both the crossvalidated *Q*2 values of our PLS models (Table 1) and the small-scale external validation, our methodology shows great potential for accurately predicting important characteristics of samples from their protein expression profiles. The large number of variables analysed in this study contributes to stabilise the multivariate models although the number of observations is small. This has been shown by extensive Monte Carlo simulations of data matrices with different combinations of observations and variables and where the conclusion was that for PLS the probability of a chance correlation is decreasing with increasing number of variables (Wakeling and Morris, 1993). However, in order to create robust and predictive models that could be applied to new samples this kind of analysis needs to be performed on significantly larger data sets, covering a wider array of instrumental and experimental factors. It is possible that the specificity of the current study could have been improved by employing a more selective approach to tissue sampling, such as laser capture micro dissection, to analyse only the tumour cell compartment of the tumour, rather than mixtures of tumour and stroma cells, as in the present study. However, a tumour consists of two interacting cellular compartments and in our opinion it is important to study both compartments in the search for tumour biomarkers. Important biomarkers may be expressed by tumour stromal cells, such as endothelium, under the influence of tumour cells.

In conclusion, using MS and multivariate analysis we have demonstrated differences in protein expression pattern between experimental rat glioma and normal brain. We found variations over time in protein expression concomitant with the tumour progression *in vivo* and, most noteworthy, that irradiation of the tumours induced changes in protein expression that allowed the irradiated tumours to be clearly distinguished from the nontreated tumours. We believe that the 77 identified peaks discriminating irradiated from nonirradiated tumours may include useful markers for the efficacy of radiation treatment of malignant glioma.

## Figures and Tables

**Table 1:** Statistical details of each separate PLS model underlying the localisation of the spectral regions of interest.

**Data set**	**A**	**R2X(cum)**	**R2Y(cum)**	**Q2Y(cum)**
Low Sup	2	0.636	0.906	0.818
High Sup	2	0.818	0.881	0.788
Low Pel	2	0.531	0.888	0.713
High Pel	2	0.530	0.968	0.902

A=number of components; High=high mass range (20–50 kDa); Low=low mass range (2–20 kDa); Pel=pellet fraction; PLS=partial least squares; Sup=supernatant fraction.

**Table 2:** Peaks within regions of interest, whose intensity significantly changed after radiotherapy

**Supernatant fraction**				
**Bin (*m/z*)**	**Mean (Ctrl)**	**Mean (Rt)**	**Sign.**	**Test**
*Low mass*				
2612.0	7.81E-04	1.70E-03	^***^	t
2671.5	4.13E-04	7.03E-04	^**^	t
2794.0	2.31E-03	5.83E-04	^***^	t
2909.5	6.16E-04	2.15E-04	^***^	t
2979.5	7.87E-04	4.12E-04	^**^	t
3119.5	7.64E-04	1.86E-04	^***^	t
3263.0	1.21E-03	6.70E-04	^*^	t
3333.0	2.59E-03	1.49E-03	^*^	t
3382.0	3.15E-04	6.93E-04	^***^	t
3427.5	7.97E-04	4.67E-04	^**^	t
3826.5	4.00E-03	1.90E-03	^***^	t
3903.5	6.70E-04	2.98E-04	^***^	t
4411.0	3.01E-03	1.02E-03	^*^	lme
4442.5	1.24E-03	2.07E-03	^***^	t
4502.0	1.22E-03	7.73E-04	^***^	t
4551.0	4.26E-04	8.24E-04	^***^	t
4617.5	4.04E-04	2.17E-04	^***^	t
5006.0	4.31E-04	7.13E-04	^*^	lme
5072.5	1.14E-03	6.27E-04	^***^	t
5226.5	9.68E-04	5.41E-04	^***^	t
5377.0	6.96E-04	1.04E-03	^*^	lme
5667.5	3.15E-04	9.62E-05	^*^	lme
5695.5	3.81E-04	1.47E-04	^**^	lme
6049.0	3.40E-04	1.08E-04	^***^	t
6178.5	1.66E-03	8.28E-04	^***^	t
6231.0	4.70E-04	2.49E-04	^***^	t
6535.5	7.68E-04	3.66E-04	^**^	lme
7473.5	1.39E-03	3.66E-04	^***^	t
10147.5	3.00E-03	1.48E-03	^**^	lme
10357.5	4.41E-04	2.36E-04	^**^	lme
13063.0	6.12E-04	2.56E-04	^**^	lme

*High mass*				
20054	2.74E-04	9.99E-05	^***^	t
20174	2.68E-04	1.00E-04	^*^	lme
20378	4.60E-04	2.03E-04	^*^	lme
20768	3.06E-04	1.42E-04	^*^	lme
20870	2.83E-04	1.10E-04	^***^	t
20882	2.81E-04	1.11E-04	^*^	lme
26198	5.11E-04	2.63E-04	^*^	lme
26216	5.18E-04	2.62E-04	^*^	lme
26630	2.55E-04	1.25E-04	^*^	lme
26846	2.58E-04	1.30E-04	^*^	lme
28346	2.56E-04	1.64E-04	^***^	t
29018	3.16E-04	2.40E-04	^*^	lme

**Pellet fraction**				
*Low mass*				
2916.5	2.09E-03	6.91E-04	**	t
3046.0	6.54E-04	2.49E-04	**	t
3081.0	3.28E-03	1.13E-03	**	t
3200.0	1.66E-03	5.37E-04	***	t
3214.0	4.75E-04	1.97E-04	**	t
3501.0	2.38E-03	1.45E-03	*	t
6752.5	1.06E-03	6.05E-04	**	t
7256.5	2.00E-03	1.08E-03	*	t
7284.5	1.78E-03	9.36E-04	**	t
7508.5	2.10E-03	4.64E-03	*	lme
7652.0	3.06E-04	7.61E-04	**	lme
7718.5	2.42E-04	5.73E-04	**	lme
8152.5	6.24E-04	1.27E-03	**	lme
8677.5	8.06E-04	5.39E-04	*	t
9087.0	8.48E-04	5.59E-04	*	t
9696.0	1.12E-03	1.39E-03	*	t
9804.5	1.77E-03	3.36E-03	*	lme
*High mass*				
20588	9.29E-04	2.94E-04	***	t
20756	8.15E-04	3.12E-04	*	lme
20960	5.35E-04	1.36E-04	***	t
21320	3.23E-04	1.28E-04	**	t
24656	3.35E-04	4.15E-04	*	t
24668	3.33E-04	4.13E-04	*	t
24686	3.20E-04	4.10E-04	*	t
24860	2.33E-04	3.70E-04	***	t
25016	1.69E-04	2.92E-04	***	t
25046	1.75E-04	2.92E-04	**	t
25088	1.61E-04	3.15E-04	***	t
25100	1.59E-04	3.11E-04	***	t
25196	2.41E-04	3.54E-04	*	t
28136	9.22E-04	7.93E-04	*	t
28694	3.68E-04	2.97E-04	*	t
44558	3.31E-04	4.79E-04	**	t
44600	3.36E-04	4.84E-04	**	t

Test=statistical test used to test whether the differences in intensity of the respective peak between groups were significant or not. lme=linear mixed effects; *t*=Students' *t*-test. ^*^The *Bin (m/z)* column lists the beginning of the bin interval in which each peak was found (the bin sizes for the low mass and high mass regions are 3.5 and 7 Da, respectively). ^†^The *Mean (Ctrl)* and *Mean (Rt)* columns present each peak's normalised mean intensity in the untreated control group and the radiotherapy-treated group, respectively. ^‡^The level of significance is indicated by asterisks, as follows:

*** *P*<0.001

** *P*<0.01

* *P*⩽0.05.

## References

[bib1] Adam BL, Qu Y, Davis JW, Ward MD, Clements MA, Cazares LH, Semmes OJ, Schellhammer PF, Yasui Y, Feng Z, Wright Jr GL (2002) Serum protein fingerprinting coupled with a pattern-matching algorithm distinguishes prostate cancer from benign prostate hyperplasia and healthy men. Cancer Res 62: 3609–3614 12097261

[bib2] Ball G, Mian S, Holding F, Allibone RO, Lowe J, Ali S, Li G, McCardle S, Ellis IO, Creaser C, Rees RC (2002) An integrated approach utilizing artificial neural networks and SELDI mass spectrometry for the classification of human tumours and rapid identification of potential biomarkers. Bioinformatics 18: 395–404 11934738 10.1093/bioinformatics/18.3.395

[bib3] Banez LL, Prasanna P, Sun L, Ali A, Zou Z, Adam BL, McLeod DG, Moul JW, Srivastava S (2003) Diagnostic potential of serum proteomic patterns in prostate cancer. J Urol 170: 442–446 12853795 10.1097/01.ju.0000069431.95404.56

[bib4] Bergenheim AT, Elfverson J, Gunnarsson PO, Edman K, Hartman M, Henriksson R (1994) Cytotoxic effect and uptake of estramustine in a rat glioma model. Int J Oncol 5: 293–299 21559588 10.3892/ijo.5.2.293

[bib5] Burnham AJ, MacGregor JF, Viveros R (1999) A statistical framework for multivariate latent variable regression methods based on maximum likelihood. J Chemometr 13: 49–65

[bib6] Burnham AJ, Viveros R, MacGregor JF (1996) Frameworks for latent variable multivariate regression. J Chemometr 10: 31–45

[bib7] Cheung PK, Woolcock B, Adomat H, Sutcliffe M, Bainbridge TC, Jones EC, Webber D, Kinahan T, Sadar M, Gleave ME, Vielkind J (2004) Protein profiling of microdissected prostate tissue links growth differentiation factor 15 to prostate carcinogenesis. Cancer Res 64: 5929–5933 15342369 10.1158/0008-5472.CAN-04-1216

[bib8] Costello A, Shallice T, Gullan R, Beaney R (2004) The early effects of radiotherapy on intellectual and cognitive functioning in patients with frontal brain tumours: the use of a new neuropsychological methodology. J Neurooncol 67: 351–359 15164992 10.1023/b:neon.0000024239.99645.42

[bib9] Eriksson L, Antti H, Gottfries J, Holmes E, Johansson E, Lindgren F, Long I, Lundstedt T, Trygg J, Wold S (2004) Using chemometrics for navigating in the large data sets of genomics, proteomics, and metabonomics (GPM). Anal Bioanal Chem 380: 419–429 15448969 10.1007/s00216-004-2783-y

[bib10] Furuta M, Weil RJ, Vortmeyer AO, Huang S, Lei J, Huang TN, Lee YS, Bhowmick DA, Lubensky IA, Oldfield EH, Zhuang Z (2004) Protein patterns and proteins that identify subtypes of glioblastoma multiforme. Oncogene 23: 6806–6814 15286718 10.1038/sj.onc.1207770

[bib11] Gadducci A, Cosio S, Carpi A, Nicolini A, Genazzani AR (2004) Serum tumor markers in the management of ovarian, endometrial and cervical cancer. Biomed Pharmacother 58: 24–38 14739059 10.1016/j.biopha.2003.11.003

[bib12] Garthwaite PH (1994) An interpretation of partial least-squares. J Am Stat Assoc 89: 122–127

[bib13] Iwadate Y, Sakaida T, Hiwasa T, Nagai Y, Ishikura H, Takiguchi M, Yamaura A (2004) Molecular classification and survival prediction in human gliomas based on proteome analysis. Cancer Res 64: 2496–2501 15059904 10.1158/0008-5472.can-03-1254

[bib14] Johansson D, Lindgren P, Berglund A (2003) A multivariate approach applied to microarray data for identification of genes with cell cycle-coupled transcription. Bioinformatics 19: 467–473 12611801 10.1093/bioinformatics/btg017

[bib15] Johansson M, Bergenheim AT, Widmark A, Henriksson R (1999) Effects of radiotherapy and estramustine on the microvasculature in malignant glioma. Br J Cancer 80: 142–148 10389990 10.1038/sj.bjc.6690333 PMC2362991

[bib16] Johansson M, Henriksson R, Bergenheim AT, Koskinen LO (2000) Interleukin-2 and histamine in combination inhibit tumour growth and angiogenesis in malignant glioma. Br J Cancer 83: 826–832 10952789 10.1054/bjoc.2000.1354 PMC2363533

[bib17] Jolliffe IT (1986) Principal Component Analysis. Springer Series in Statistics. Springer-Verlag: New York

[bib18] Kleihues P, Ohgaki H (1997) Genetics of glioma progression and the definition of primary and secondary glioblastoma. Brain Pathol 7: 1131–1136

[bib19] Koopmann J, Zhang Z, White N, Rosenzweig J, Fedarko N, Jagannath S, Canto MI, Yeo CJ, Chan DW, Goggins M (2004) Serum diagnosis of pancreatic adenocarcinoma using surface-enhanced laser desorption and ionization mass spectrometry. Clin Cancer Res 10: 860–868 14871961 10.1158/1078-0432.ccr-1167-3

[bib20] Krause DS, Van Etten RA (2005) Tyrosine kinases as targets for cancer therapy. N Engl J Med 353: 172–187 16014887 10.1056/NEJMra044389

[bib21] Liu J, Zheng S, Yu JK, Zhang JM, Chen Z (2005) Serum protein fingerprinting coupled with artificial neural network distinguishes glioma from healthy population or brain benign tumor. J Zhejiang Univ Sci 6: 4–10 10.1631/jzus.2005.B0004 PMC1390751 15593384

[bib22] Melle C, Kaufmann R, Hommann M, Bleul A, Driesch D, Ernst G, von Eggeling F (2004) Proteomic profiling in microdissected hepatocellular carcinoma tissue using ProteinChip technology. Int J Oncol 24: 885–891 15010826

[bib23] Merchant M, Weinberger SR (2000) Recent advancements in surface-enhanced laser desorption/ionization-time of flight-mass spectrometry. Electrophoresis 21: 1164–1177 10786889 10.1002/(SICI)1522-2683(20000401)21:6<1164::AID-ELPS1164>3.0.CO;2-0

[bib24] Petricoin EF, Ardekani AM, Hitt BA, Levine PJ, Fusaro VA, Steinberg SM, Mills GB, Simone C, Fishman DA, Kohn EC, Liotta LA (2002) Use of proteomic patterns in serum to identify ovarian cancer. Lancet 359: 572–577 11867112 10.1016/S0140-6736(02)07746-2

[bib25] Poon TC, Yip TT, Chan AT, Yip C, Yip V, Mok TS, Lee CC, Leung TW, Ho SK, Johnson PJ (2003) Comprehensive proteomic profiling identifies serum proteomic signatures for detection of hepatocellular carcinoma and its subtypes. Clin Chem 49: 752–760 12709366 10.1373/49.5.752

[bib26] Reyzer ML, Caldwell RL, Dugger TC, Forbes JT, Ritter CA, Guix M, Arteaga CL, Caprioli RM (2004) Early changes in protein expression detected by mass spectrometry predict tumor response to molecular therapeutics. Cancer Res 64: 9093–9100 15604278 10.1158/0008-5472.CAN-04-2231

[bib27] Reyzer ML, Caprioli RM (2005) MALDI mass spectrometry for direct tissue analysis: a new tool for biomarker discovery. J Proteome Res 4: 1138–1142 16083264 10.1021/pr050095+

[bib28] Roblick UJ, Hirschberg D, Habermann JK, Palmberg C, Becker S, Kruger S, Gustafsson M, Bruch HP, Franzen B, Ried T, Bergmann T, Auer G, Jornvall H (2004) Sequential proteome alterations during genesis and progression of colon cancer. Cell Mol Life Sci 61: 1246–1255 15141310 10.1007/s00018-004-4049-4 PMC11138807

[bib29] Schwartz SA, Weil RJ, Johnson MD, Toms SA, Caprioli RM (2004) Protein profiling in brain tumors using mass spectrometry: feasibility of a new technique for the analysis of protein expression. Clin Cancer Res 10: 981–987 14871976 10.1158/1078-0432.ccr-0927-3

[bib30] Somorjai RL, Dolenko B, Baumgartner R (2003) Class prediction and discovery using gene microarray and proteomics mass spectroscopy data: curses, caveats, cautions. Bioinformatics 19: 1484–1491 12912828 10.1093/bioinformatics/btg182

[bib31] Stupp R, Mason WP, van den Bent MJ, Weller M, Fisher B, Taphoorn MJ, Belanger K, Brandes AA, Marosi C, Bogdahn U, Curschmann J, Janzer RC, Ludwin SK, Gorlia T, Allgeier A, Lacombe D, Cairncross JG, Eisenhauer E, Mirimanoff RO (2005) Radiotherapy plus concomitant and adjuvant temozolomide for glioblastoma. N Engl J Med 352: 987–996 15758009 10.1056/NEJMoa043330

[bib32] Swennen MH, Bromberg JE, Witkamp TD, Terhaard CH, Postma TJ, Taphoorn MJ (2004) Delayed radiation toxicity after focal or whole brain radiotherapy for low-grade glioma. J Neurooncol 66: 333–339 15015665 10.1023/b:neon.0000014518.16481.7e

[bib33] Trygg J (2002) O2-PLS for qualitative and quantitative analysis in multivariate calibration. J Chemometr 16: 283–293

[bib34] Vlahou A, Laronga C, Wilson L, Gregory B, Fournier K, McGaughey D, Perry RR, Wright Jr GL, Semmes OJ (2003) A novel approach toward development of a rapid blood test for breast cancer. Clin Breast Cancer 4: 203–209 14499014 10.3816/cbc.2003.n.026

[bib35] Wakeling IN, Morris JJ (1993) A test of significance for partial least-squares regression. J Chemometr 7: 291–304

[bib36] Ware ML, Berger MS, Binder DK (2003) Molecular biology of glioma tumorigenesis. Histol Histopathol 18: 207–216 12507300 10.14670/HH-18.207

[bib37] Wiesner A (2004) Detection of tumor markers with ProteinChip((R)) technology. Curr Pharm Biotechnol 5: 45–67 14965209 10.2174/1389201043489675

[bib38] Wold H (1966) Estimation of principal components and related models by iterative least squares. In Multivariate Analysis, Krishnaiah PR (ed) pp 391–420. Academic press: New York

[bib39] Wold S, Albano C, Dunn III WJ, Esbensen K, Hellberg S, Johansson E, Sjöström M (1982) Multivariate analytical chemical data evaluation using SIMCA and MACUP. In Scientific Symposium: Pattern Recognition in Analytical Chemistry, pp 157–188. Mátrafüred, Hungary

[bib40] Wold S, Sjostrom M, Eriksson L (2001) PLS-regression: a basic tool of chemometrics. Chemometr Intell Lab 58: 109–130

[bib41] Workman P, Twentyman P, Balkwill F, Balmain A, Chaplin D, Double J, Embleton J, Newell D, Raymond R, Stables J, Stephens T, Wallace J (1998) United Kingdom Co-Ordinating Committee on Cancer Research (UKCCCR) Guidelines for the Welfare of Animals in Experimental Neoplasia (Second Edition). Br J Cancer 77: 1–10 10.1038/bjc.1998.1 PMC2151254 9459138

[bib42] Zhang R, Tremblay TL, McDermid A, Thibault P, Stanimirovic D (2003) Identification of differentially expressed proteins in human glioblastoma cell lines and tumors. Glia 42: 194–208 12655603 10.1002/glia.10222

